# E-Peptides Control Bioavailability of IGF-1

**DOI:** 10.1371/journal.pone.0051152

**Published:** 2012-12-10

**Authors:** Marianne Smedegaard Hede, Ekaterina Salimova, Agnieszka Piszczek, Emarald Perlas, Nadine Winn, Tommaso Nastasi, Nadia Rosenthal

**Affiliations:** 1 European Molecular Biology Laboratory (EMBL)-Mouse Biology Unit, Monterotondo, Rome, Italy; 2 Australian Regenerative Medicine Institute, EMBL Australia/Monash University, Melbourne, Australia; 3 National Heart and Lung Institute, Imperial College London, London, United Kingdom; University of Rome La Sapienza, Italy

## Abstract

Insulin-like growth factor 1 (IGF-1) is a potent cytoprotective growth factor that has attracted considerable attention as a promising therapeutic agent. Transgenic over-expression of IGF-1 propeptides facilitates protection and repair in a broad range of tissues, although transgenic mice over-expressing IGF-1 propeptides display little or no increase in IGF-1 serum levels, even with high levels of transgene expression. IGF-1 propeptides are encoded by multiple alternatively spliced transcripts including C-terminal extension (E) peptides, which are highly positively charged. In the present study, we use decellularized mouse tissue to show that the E-peptides facilitate *in vitro* binding of murine IGF-1 to the extracellular matrix (ECM) with varying affinities. This property is independent of IGF-1, since proteins consisting of the E-peptides fused to relaxin, a related member of the insulin superfamily, bound equally avidly to decellularized ECM. Thus, the E-peptides control IGF-1 bioavailability by preventing systemic circulation, offering a potentially powerful way to tether IGF-1 and other therapeutic proteins to the site of synthesis and/or administration.

## Introduction

Insulin-like Growth Factor-1 (IGF-1) is a potent peptide factor involved in a broad range of tissue processes including cell growth and survival, proliferation, differentiation and metabolism, but the molecular basis of these diverse functions is not well understood. In the adult mammal, IGF-1 is synthesized predominately in the liver, and acts as a systemic growth factor, playing important roles in both normal and neoplastic growth [1]. IGF-1 is also produced in extrahepatic tissues where it plays a predominantly autocrine/paracrine role in local processes. Despite a significant reduction of serum IGF-1 peptide levels in mice where the *Igf-1* gene was deleted conditionally in the liver, other parameters were largely normal, indicating that locally synthesized IGF-1 can support normal postnatal growth and development [2].

**Figure 1:**
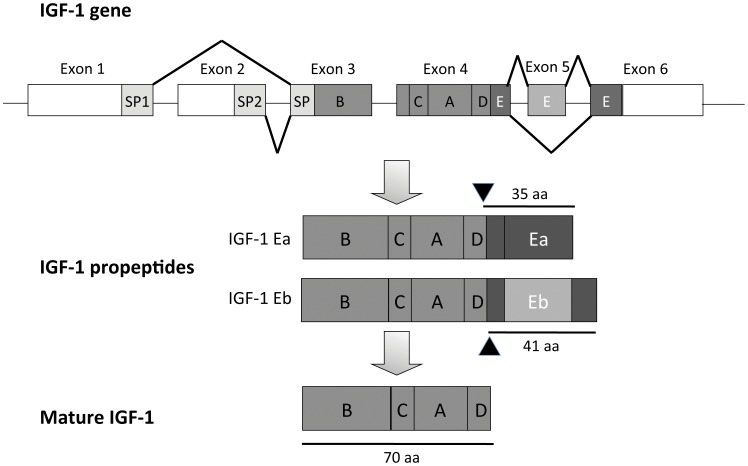
Structure of the rodent IGF-1 gene. Exons 1 and 2 are transcribed from different promoters. Differential splicing gives rise to two different signal peptides (SP1 and SP2), which include a common C-terminal sequence encoded by Exon 3. Exon 3 also encodes the N-terminal part of the mature IGF-1 B chain. Exon 4 encodes the remaining mature IGF-1 protein (B,C,A and D chains), and also encodes the common N-terminal sequence of the E-peptides. Differential splicing excluding Exon 5 gives rise to the IGF-1Ea propeptide, or a longer IGF-1Eb propeptide when Exon 5 is included. Protease cleavage (arrowheads) removes the E peptides to produce the mature IGF-1 protein.

The diversity of IGF-1 actions may derive from the existence of several different isoforms that differ from one another due to alternative splicing of the initial transcript [3], [4]. The single copy *Igf-1* gene locus encodes multiple pre-propeptide precursors in which the mature protein is flanked by variable N-terminal signal peptides and C-terminal extension (E) peptides. In the mouse, the *Igf-1* gene encodes four main pre-propeptides, combining signal peptides (SP1 or SP2) with Ea or Eb extension peptides (Figure 1). As these pre-propeptides all undergo post-translational processing to generate the same mature 70 aa IGF-1 protein, the specific roles of E-peptides in IGF-1 biology remain unclear. One of the isolated E-peptides (Eb, renamed MGF) has been reported to increase the regenerative capability of skeletal muscle, play a neuroprotective role against ischemia, and facilitate the actions of IGF-1 to improve cardiac function and mobilize resident stem cell populations [5], [6], [7]. Other studies suggest that E-peptides are not required for IGF-1 secretion but increase cell entry of IGF-1 from the media [8].

Transgenic studies have shed further light on the role of E-peptides. IGF-1Ea propeptide provided as a muscle-specific transgene results in muscle hypertrophy and enhances regeneration after injury [9], [10], [11], reducing inflammation and fibrosis [12]. This phenotype is unaffected by the choice of N-terminal signal peptide [13] but is not recapitulated by a muscle-specific transgene encoding IGF-1 lacking an E-peptide moiety, which produces no local effects but instead significantly increases serum IGF-1 levels [14]. The dramatic phenotypes resulting from supplemental tissue-specific IGF-1Ea transgene expression in other tissues such as heart [15] and skin [16], with no increase in circulating IGF-1 levels, suggests a role for E-peptides in local IGF-1 action and retention of IGF-1 in the tissue of synthesis.

To directly test this hypothesis, we analyzed transgenic mice expressing each of the four major IGF-1 prepropeptides under the control of a muscle-specific regulatory element and assessed the presence of transgene products in circulation. We investigated the relative retention of various IGF-1 moieties on decellularized tissue preparations. Here we show that both IGF-1Ea and IGF-1Eb propeptides bind extracellular matrix with significantly higher affinity than does mature IGF-1. E-peptide-mediated ECM binding is independent of the mature IGF-1 sequence, since they also facilitate ECM binding when fused to relaxin, another insulin-related factor. These results suggest a novel role for E-peptides in controlling bioavailability of IGF-1, by tethering the protein to the site of synthesis through enhanced affinity for the extracellular matrix.

## Results

### Transgenic IGF-1 Propeptides are Retained in Skeletal Muscle

**Figure 2:**
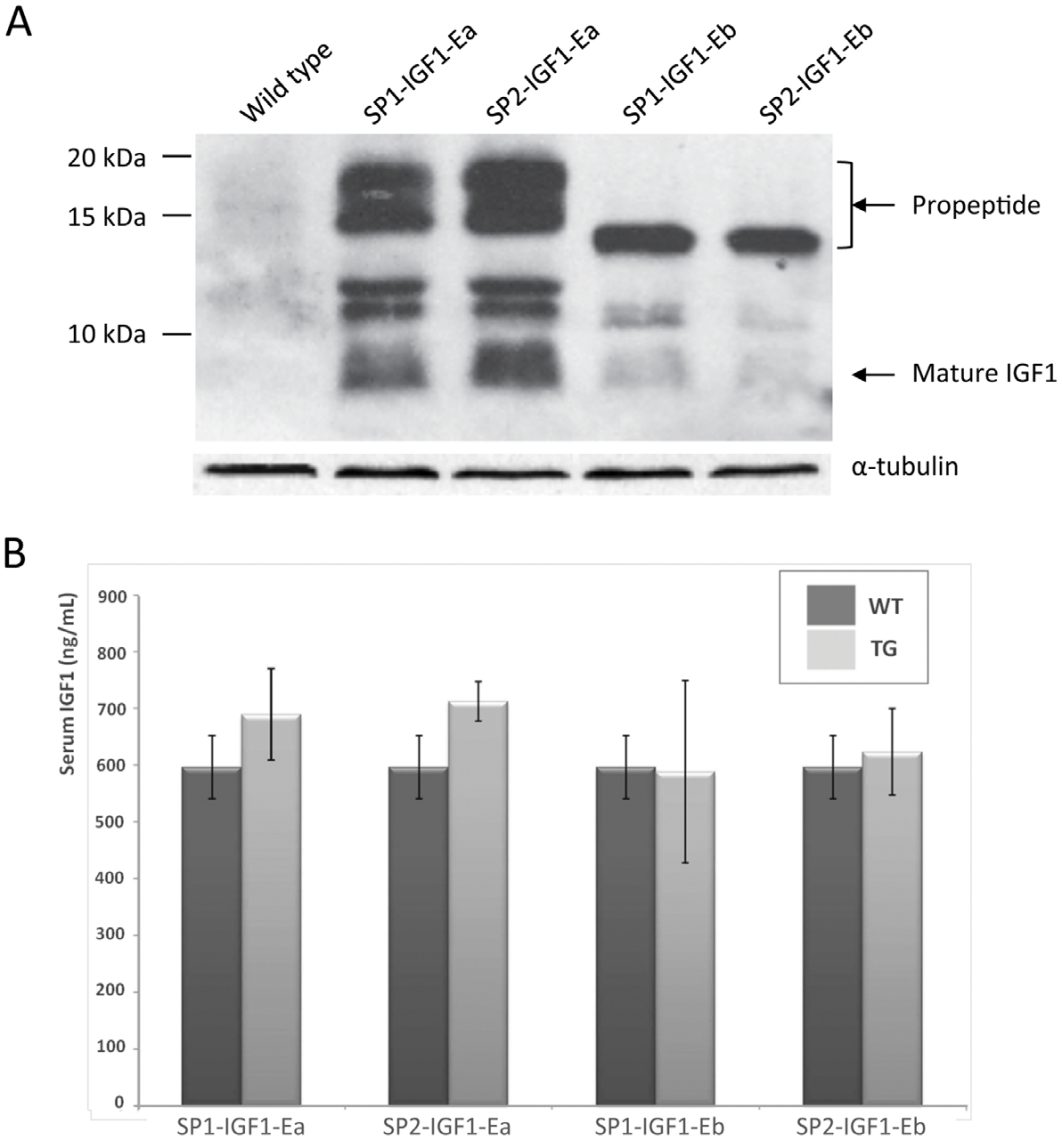
IGF-1 expression and secretion in transgenic animals. **A)** Western blot analysis of IGF-1 transgene levels in quadriceps muscle of 3 months old male mice. **B)** Total IGF-1 levels in the blood serum of 3 months old transgenic male mice compared to WT littermates as determined by ELISA.

Transgenic mice were generated with the four main IGF-1 splicing variants, combining the two signal peptides and two E peptides (Figure 1), controlled by the fast IIB muscle fiber-specific myosin light chain promoter (MLC1/3) and enhancer ([11], which drive expression exclusively in skeletal muscle (See Materials and Methods section). Western blot analysis of quadriceps muscles showed comparable IGF-1 protein levels in the four transgenic lines, which did not reflect variable transcript levels as revealed by Northern blot (Figure S1) suggesting that isoform concentration may be controlled post-transcriptionally. The majority of the transgenic protein was unprocessed or partially processed (Figure 2A). Additional bands likely reflect differential glycosylation states, since the rodent Ea-peptide contains two N-linked glycosylation sites that are absent in the Eb-peptide [17], [18]. Total serum analysis revealed no increase in IGF-1 levels in mice carrying IGF-1Eb transgenes and only a slight increase in mice carrying IGF-1Ea transgenes (16±13.5% and 19±6% respectively) (Figure 2B). Thus the majority of both IGF-1Ea and IGF-1Eb transgenic products are retained in the tissue of synthesis as propeptides. On the contrary, transgenic mice expressing mature IGF-1 (lacking E-peptide) driven by rat skeletal α-actin promoter showed increased levels of systemic IGF-1 [14], [19], implicating the E peptide moiety in the retention of IGF-1 at the site of synthesis.

### E-peptides are Positively Charged and Promote Binding to Negatively Charged Surfaces

Examination of the E-peptide primary sequences revealed an unusual proportion of basic amino acid residues, conferring the peptides with a high positive charge at physiological pH (Table 1). The extracellular matrix (ECM) is rich in negatively charged polysaccharides and sulfated components, which modulate the diffusion of secreted proteins [20]. To test the hypothesis that the E-peptide moieties might bind to negatively charged molecules in the ECM, we generated IGF-1 propeptides with appropriate post-translational modifications by transfecting HEK 293 cells with cDNA expression constructs encoding Class 1 signal peptide (SP1) and the mature mouse IGF-1 (IGF-1 Stop), IGF-1Ea, or IGF-1Eb propeptides. In the latter two constructs, mutations in the E-peptide cleavage sites (arrowheads in Figure 1) were introduced to prevent proteolytic removal of E peptides (see Materials and Methods section). These constructs are thereafter denoted as cleavage deficient (IGF-1EaCD and IGF-1EbCD).

**Table 1:** Length (amino acids), Isoelectric Point (IP), and calculated charge at pH7 of human (h) (rows 1–5) and murine (mu) (rows 6–10) IGF-1 related peptides.

Peptide	Length (aa)	IP	Charge at pH7
**Mature hIGF-1**	70	7.76	0.71
**hEa**	35	11.48	6.93
**hEc**	40	11.42	8.85
**hIGF1-Ea**	105	9.47	7.88
**hIGF1-Ec**	110	9.65	9.80
**Mature muIGF-1**	70	8.31	1.71
**muEa**	35	11.48	6.93
**muEb**	41	11.74	9.93
**muIGF1-Ea**	105	9.60	8.88
**muIGF1-Eb**	111	9.88	11.88

Rows 1 and 6: mature IGF-1; rows 4,5,9,10: propeptides; rows 2,3,7,8: E-peptides alone.

**Figure 3:**
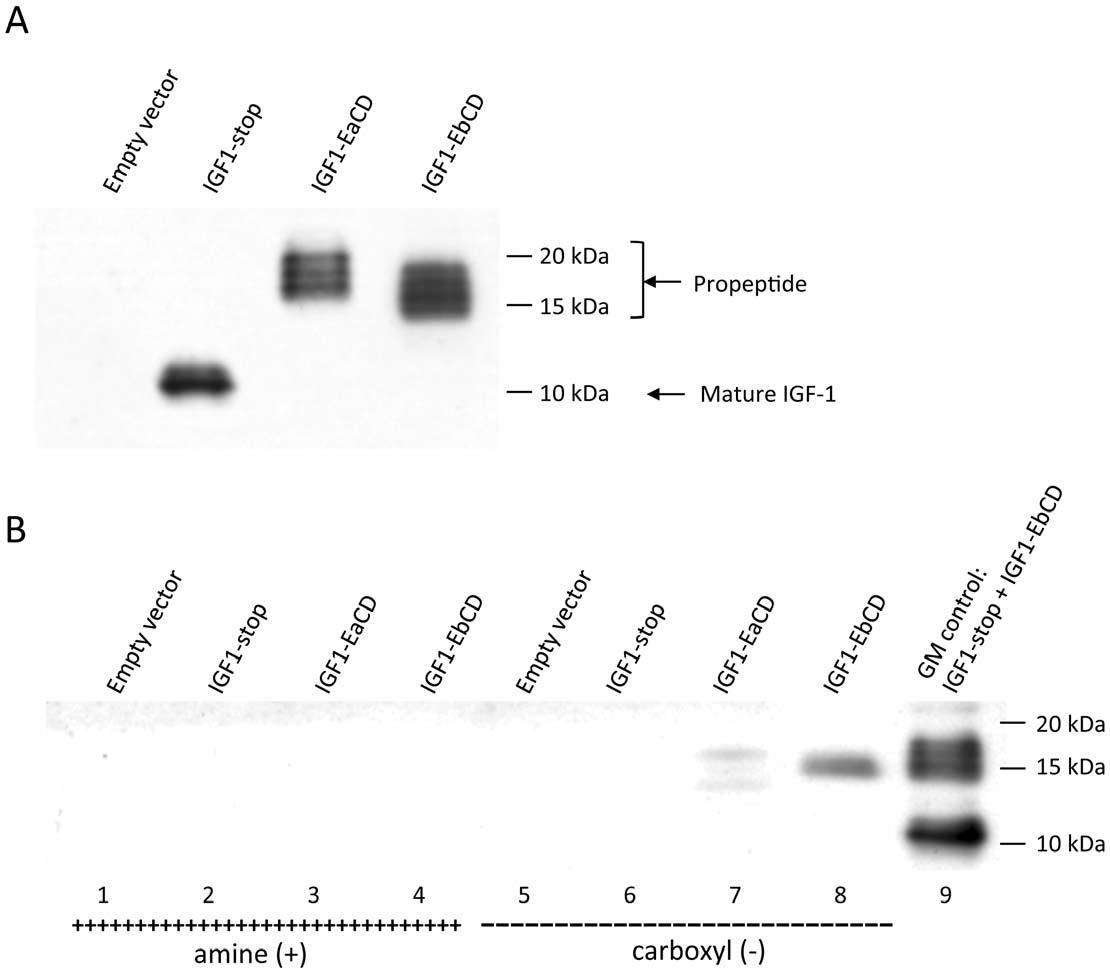
E-peptides promote binding of IGF-1 to negatively charged surfaces. **A)** Growth medium (10 uL) from transiently transfected HEK 293 cells (IGF-1 levels normalised to 200 ng/mL). **B)** Binding of IGF-1 propeptides to positively (amine) (lanes 2–4) and negatively (carboxyl) (lanes 6–8) charged tissue culture plates. The control lane (9) is a mixture of growth media from IGF-1-stop and IGF-1EbCD transfected cells.

To assess the binding capacity of IGF-1 propeptides, we exploited the charged surfaces of different tissue culture plates. Growth media containing IGF-1-stop, IGF-1EaCD or IGF-1EbCD secreted peptides (Figure 3A), normalized to 200 ng/mL of IGF-1, was added directly into the wells of negatively (carboxyl) and positively (amine) charged tissue culture plates (BD PureCoat), incubated, washed and extracted as described in the Materials and Methods section. Western blot analysis showed that only E-peptide-containing IGF-1 propeptides were able to bind to the negatively charged surfaces (Figure 3B, lanes 6–8), while no binding to positively charged surfaces was detected (Figure 3B, lanes 2–4). IGF-1Eb showed stronger affinity to the negatively charged surface then IGF-1Ea (Figure 3B, lanes 7 and 8). No degradation during incubation was observed (data not shown).

### E peptides Confer IGF-1 Binding to Heparin Agarose

**Figure 4:**
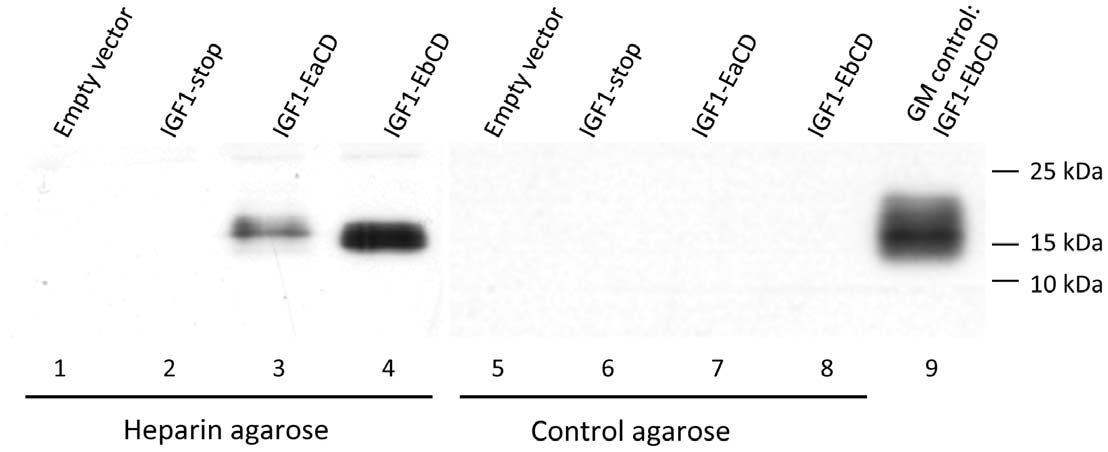
E-peptides bind heparin-agarose. Binding of IGF-1 isoforms to heparin coated agarose beads (lanes 2–4) and control agarose beads (lanes 6–8). The control lane (9) is the growth medium from IGF-1EbCD transfected cells.

Heparin, a highly sulfated glycosaminoglycan and a major component of ECM, is known to have the highest negative charge density of any known biological molecule [21], [22]. To assess the binding of IGF-1EaCD and IGF-1EbCD propeptides heparin-coated agarose beads were incubated with conditioned growth medium (see Figure 3A) and then washed and extracted as described in Materials and Methods. Western Blot analysis revealed that only IGF-1 containing E-peptides bound to the heparin beads (Figure 4) with IGF-1Eb showing stronger binding than IGF-1-Ea (Figure 4, lanes 3 and 4). No binding to control agarose beads was observed (Figure 4, lanes 6–8).

### IGF-1 E-peptide Moieties Promote Binding to Extracellular Matrix

**Figure 5:**
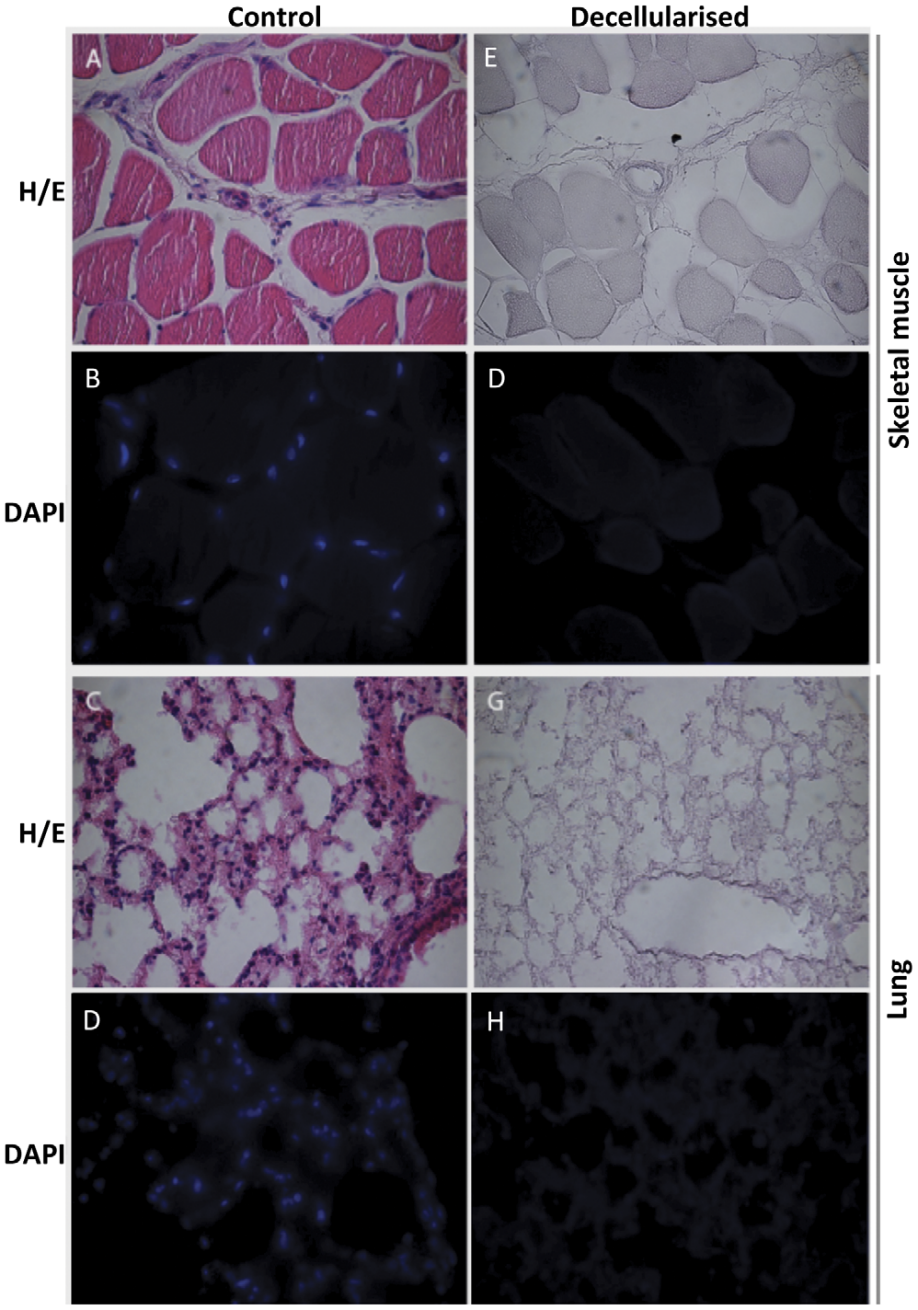
Preparation of decellularized tissue as ECM substrate. Sections of paraffin imbedded control (non-decellularized) skeletal muscle and lung tissue (A-D) or decellularized skeletal muscle and lung tissue (E-H). The sections were stained with hematoxylin/eosin (H/E) or DAPI as indicated.

**Figure 6:**
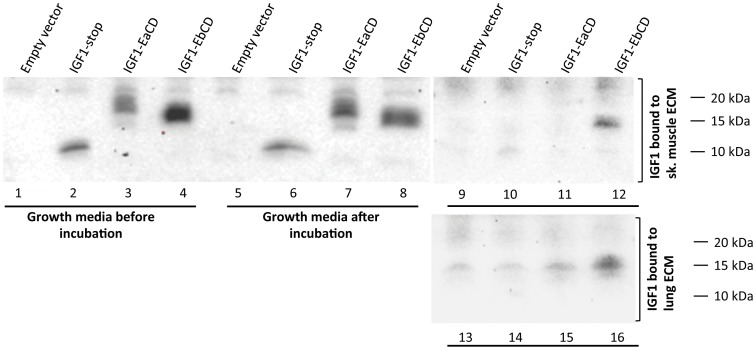
IGF-1 propeptides bind to the ECM. Western blot analysis of IGF-1 binding. Lanes 1–4: growth media from HEK 293 cells transfected with IGF-1 expression plasmids encoding either the mature peptide (lane 2) or cleavage deficient IGF-1 propeptides (lanes 3 and 4). Lanes 5–8: same growth media after incubation with decellularizsed tissue. Lanes 9–12: IGF-1 binding to decellularized lung tissue. Lanes 13–16: IGF-1 binding to decellularizsed skeletal muscle tissue.

To obtain a biologically relevant substrate for studying binding of secreted peptides to the ECM, various soft murine tissues were decellularized as described by Gillies et al [23]. This protocol avoids usage of proteases or detergents and thus results in a largely native ECM substrate with intact three-dimensional configuration. Of a range of different tissues (data not shown) skeletal muscle and lung yielded the most complete and consistent decellularization. To validate the integrity of the preparation and lack of residual cellular material, decellularized tissue was paraffin imbedded, sectioned, and stained with either hematoxylin/eosin or with DAPI. As shown in Figure 5, both lung tissue (Figure 5C,D) and quadriceps muscle (Figure 5A,B) were effectively decellularized with no cellular debris or DNA remaining. As seen in Figure 6, decellularized lung and skeletal muscle tissues were incubated in the conditioned growth media from transiently transfected HEK293 cell cultures (see Figure 3A). After one hour incubation at 37°C no major degradation of IGF-1 peptides was observed (Figure 6, lanes 2–4 vs lanes 6–8). After washing and extraction (see Materials and Methods), Western blot analysis clearly showed that IGF-1EaCD and IGF-1EbCD adhered to the decellularized matrix more avidly than did the mature IGF-1 protein (IGF-1-stop), with IGF-1Eb propeptide having the highest ECM binding affinity (Figure 6, lanes 10–12 and 14–16).

### Focal Binding of IGF-1 Propeptides to ECM

**Figure 7:**
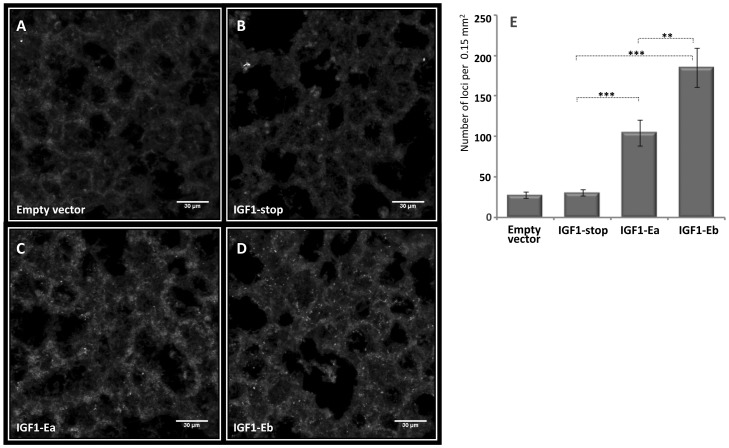
IGF-1 propeptides bind to the ECM at particular loci. A–D) Decellularized lung tissue was sectioned and incubated with growth media from HEK 293 cells transfected with IGF-1 expression plasmids (see Materials and Methods for details). Bound IGF-1 was visualized by immunostaining using anti-IGF-1 antibody. E) Quantification of the number of IGF-1 loci. Data is presented as mean (SE) for 20 biological replicates. Two stars corresponds to P<0.01, three stars correspond to P<0.001.

To further characterize the binding of IGF-1 propeptides to the ECM, decellularized lung tissue was paraffin embedded, sectioned, incubated with the conditioned growth media (Figure 3A), and subsequently stained for IGF-1 protein. As shown in Figure 7, sections incubated with IGF-1-stop displayed significantly less IGF-1 containing loci than did sections incubated with IGF-1EaCD or IGF-1EbCD. Notably, IGF-1EbCD produced more IGF-1-containing loci than did IGF-1EaCD, reflecting the higher ECM binding affinity of the Eb peptide.

### E-peptide Mediated Binding of an Unrelated Protein to the ECM

**Figure 8:**
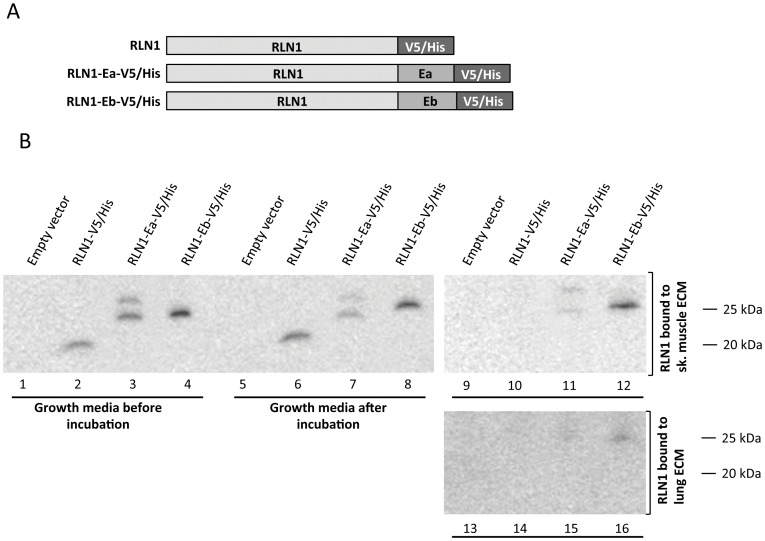
E-peptide mediated binding to the ECM is independent of IGF-1. A) Schematic representation of the three relaxin based constructs used for the experiments. Fusions of murine relaxin (RLN1)to murine Ea and Eb peptides were produced. The constructs were tagged with biochemical tags (V5 and poly-His). B) V5 Western blot analysis. Lanes 1–4: growth media from HEK 293 cells transfected with RLN1 expression plasmids. Lanes 5–8: same growth media after incubation with decellularizsed tissue. Lanes 9–12: binding of RLN1 constructs to decellularized lung tissue. Lanes 13–16: binding of RLN1 constructs decellularizsed skeletal muscle tissue.

To determine whether the E-peptide mediated binding to the ECM is independent of the core IGF-1 sequence, we fused IGF-1 E-peptides to relaxin (RLN1 propeptide), another member of the insulin superfamily. Fusion peptides contained a C-terminal V5 epitope and a polyhistidine tag for detection (V5 and His) (Figure 8). The constructs, RLN1-V5/His, RLN1-Ea-V5/His, RLN1-Eb-V5/His were expressed in transiently transfected HEK 293 cells and the conditioned media was incubated with decellularized lung tissue as described above. The extracts were analyzed by Western blot for the V5 tag. No detectable degradation during incubation was observed (lanes 2–4 vs. lanes 6–8). Comparison of lanes 2, 6and 10 shows that in the absence of E peptide, RLN1-V5 was almost completely washed away from the tissue samples during the post-binding wash steps, whereas the RLN1-Ea/b-V5 peptides were retained. As in case with IGF-1, RLN1-Eb fusion showed stronger affinity to ECM then the RLN1-Ea. This confirms that the E-peptide mediated binding of proteins to the ECM is an inherent feature of the E-peptide sequences.

## Discussion

In this study we report a novel ECM tethering function for the C-terminal IGF-1 E peptides, which presumably reflects a biological role in maintaining high local concentrations of the growth factor at the site of synthesis. The primary sequence of the *Igf-1* gene has been unevenly conserved during evolution: whereas the encoded mature IGF-1 protein only differs at a single amino acid between mouse and man, E peptide sequences are more variable across species. Despite the lack of sequence homology, E-peptides from a broad range of species all retain an unusually high basic amino acid content leading to a positive charge at physiological pH. This evolutionary conservation of charge strongly argues for its function in modulating growth factor diffusion rate and/or biological availability. Using a novel strategy involving Western blot analysis of bound moieties, we show here that IGF-1 propeptides adhere much more readily to the ECM than does mature, fully processed IGF-1, although a small percentage of mature IGF-1 also binds to the decellularized tissue, consistent with previous reports showing indirect binding of mature IGF-1 to the ECM through the IGF-1 binding proteins [24], [25]. A tethering role for IGF-1 E peptides is consistent with previous observations that transgenic mice over-expressing IGF-1 propeptides in a number of tissues do not show increased IGF-1 serum levels ([11], [15], [16], whereas mice expressing an IGF-1 transgene lacking an E-peptide display dramatically elevated serum IGF-1 [14].

The ECM is a complex mixture of fibrous proteins and proteoglycans that surrounds and supports the cells of multicellular organisms, binding circulating peptide hormones and modulating their activity [26]. In particular, heparan sulfate proteoglycan (HSPG), a prominent component of ECM, binds a wide range of growth factors and cytokines, including members of the PDGF, VEGF, EGF, FGF, TGF-β families (reviewed in [22], likely mediated by positively charged amino acid sequence motifs present in these peptides. This common feature would allow the formation of specific gradients of these factors and/or their retention at the site of synthesis. However, decellularized tissues such as the substrate used in the present study provide a near-native ECM with only very minor damages to the matrix integrity, more accurately resembling the complex mesh of the natural structure than does Matrigel, the most commonly used ECM substrate, which varies across batches (MH and ES, unpublished observations). Decellularized tissues, on the other hand, offer a new and surprisingly solid model for studying the ECM. Moreover, they have been successfully recellularized to form at least partly functional organs [27], [28], [29].

The fact that the IGF-1Eb propeptide displays higher affinity for ECM than does the IGF-1Ea propeptide may be attributed to a lower positive charge on the Ea peptide (see Table 1), as well as to preferential glycosylation of the Ea peptide that may significantly neutralize its positive charge [17]. Our preliminary data on deglycosylation of IGF-1 propeptides strongly support this hypothesis (see Figure S2). Deglycosylated IGF-1Ea showed much stronger affinity to negatively charged tissue culture surfaces, while very a modest difference was observed in case of IGF1-Eb. N-glycosylation has also been shown to modulate the circulation of other peptide hormones such as FGF and growth hormone [30], [31], but no function for glycosylation of the Ea peptide has so far been reported. It is tempting to speculate that the affinity of these positively charged peptides is modulated on one side by the degree of glycosylation, and on the other side by the composition of the ECM. The relative affinities of E peptides may therefore differ significantly from tissue to tissue. Further studies will be needed to address this hypothesis.

The difference in affinity to the ECM may underpin the different functions associated with IGF-1Ea and IGF-1Eb both *in vitro* and *in vivo* [32], [33], [34], [35]. In acute skeletal muscle injury, IGF-1Eb transcripts are initially upregulated, followed by a switch in splicing to generate IGF-1Ea transcripts. As the Eb-peptide and/or a proposed 24 amino acid Eb-derived peptide (MGF) has been reported to induce proliferation of a range of different cell-types [33], [36], [37], and to activate satellite cells independently of IGF-1 [7], its enhanced affinity to the ECM may facilitate initiation of the regenerative process followed by subsequent synthesis of IGF-1Ea, which is associated with enhanced fusion and differentiation of muscle progenitor cells.

As previously reported [11], the fast muscles of transgenic MLC/IGF-1Ea mice are heavier than those of WT littermates, with dramatic shift towards larger fast fibers and higher tetanic force. On the other hand, MLC/IGF-1Eb muscle groups do not display a significant increase in weight or fiber size, and no significant change in force values (Tonkin et al, 2012, manuscript in preparation). By contrast, both transgenes affected the regenerative capacity, albeit to different extents: MLC/IGF-1Ea transgenic muscles regenerated faster with an absence of inflammatory cells and fibrotic tissue, a significant increase in the CSA of newly forming myotubes and multinucleated fibers, whereas MLC/IGF-1Eb muscles did not show an increase in the size of fibers only a mild increase in the number of regenerating fibers despite improved morphology (Tonkin et al, 2012, manuscript in preparation). These observations suggest that IGF-1Ea and IG-1Eb propeptides differently affect the regeneration process. It is therefore tempting to speculate that higher affinity of Eb peptide to ECM ensures its retention at the site of damage where its mitogenic and satellite cell activating functions aid regeneration by preparing the site of damage for the subsequent boost of IGF-1-Ea, which will then facilitate the fusion and differentiation of the activated muscle precursor cells.

Our observations also suggest an appealing pharmaceutical application for the IGF-1 E-peptides. Their inherent ability to tether payload proteins to the ECM may be used to contain therapeutics at the site of administration. The IGF-1 system itself provides a compelling example of this principle, since localized overexpression of IGF-1Ea propeptide has been shown to protect a range of different tissues against degenerative diseases without increasing systemic IGF-1 levels [36], [37], [38]. Indeed, the observation that transgenic mice overexpressing mature IGF-1 without E peptides [14] show a much higher increase in IGF-1 serum levels than those overexpressing IGF-1 propeptides [11], [15], [16] directly implicates E-peptides in controlling bioavailability of IGF-1. Our findings that the interaction between E-peptides and the ECM can tether heterologous peptides suggest that the E-peptides could be used to reduce the diffusion of administrated pharmaceuticals from the site of synthesis or delivery, thereby increasing treatment efficiency, reducing the risk of side effects, and providing a novel method for improving the efficiency and safety of peptide drugs.

## Materials and Methods

### Animals

Mice were housed in a temperature-controlled mouse facility and maintained on a 12 hour light/dark cycle with free access to chow and water. Animals were sacrificed by asphyxiation with CO_2_ and/or cervical dislocation. All animal experiments were performed at EMBL Mouse Biology Unit (Monterotondo, Italy) under the license from the Italian Ministry of Health (ref. no. 85/2010-B) in accordance with the current Italian legislation on the use of animals for research (DLgs. 116/92) and with NIH guidelines for animal care.

### Generation and Characterization of IGF-1 Transgenic Mouse Lines

SP1-IGF-1Ea ( =  MLC/mIGF-1) transgenic line has been described previously [11]. Skeletal muscle specific SP1-IGF-1Eb, SP2-IGF-1Ea and SP2-IGF-1Eb expression constructs were generated by cloning the respective mouse cDNA sequences into the skeletal muscle-specific expression cassette containing the myosin light chain (MLC) 1/3 promoter and the SV40 polyadenylation signal, followed by the MLC 1/3 enhancer sequence [39] [11], [40] (See Sup. Figure 1B). IGF-1 cDNAs were cloned by RT-PCR from mouse liver using the primers listed in Table 2. Transgenic animals were generated by pronuclear injection using FVB mice as embryo donors. Positive founders were bred to FVB wild-type mice and positive transgenic mice were selected by PCR from tail digests (for primer sequence see Table 2). Primers were designed to recognize all IGF-1 isoforms by choosing a forward primer located in exon 4 and a reverse primer located in the SV40 polyadenylation signal sequence.

**Table 2:** Sequences of primers used for cloning of cDNA of IGF-1 isoforms.

	Forward	Reverse
**Class 1 IGF-1Ea**	5′-CTCGATAACTTTGCCAGAAG-3′	5′-CCTCCTACATTCTGTAGGTCTTG-3′
**Class 1 IGF-1Eb**	5′-CTCGATAACTTTGCCAGAAG-3′	5′-CCTGCACTTCCTCTACTTGTG-3′
**Class 2 IGF-1Ea**	5′-AGTTTTGTGTTCACCTCGGCC-3′	5′-CCTCCTACATTCTGTAGGTCTTG-3′
**Class 2 IGF-1Eb**	5′-AGTTTTGTGTTCACCTCGGCC-3′	5′-CCTGCACTTCCTCTACTTGTG-3′
**Genotyping**	5′-ACTGACATGCCCAAGACTCAG-3′	5′-ATTCCACCACTGCTCCCATTC-3′

Transgenic founders were analyzed for skeletal muscle-specific expression (Figure S2A) and were selected for high but comparable transgene expression levels. One founder for each line was selected and phenotype analysis was carried out on male animals. All data was compared to the previously well-described MLC/mIGF-1 ( = SP1-IGF-1Ea) transgenic line [11]. Comparison of IGF-1 expression levels was performed by Northern Blot (Sup. Figure 2B) and by western blot (Figure S2D) analysis.

### RNA Isolation and Northern Blot Analysis

Total RNA was extracted from snap-frozen tissues using TRIzol Reagent (Invitrogen). Ten micrograms of total RNA were fractionated by electrophoresis on 1.3% agarose gels containing 2.2 M formaldehyde (Sigma) and blotted and hybridized as described previously [40].

### Generation of Expression Clones for in vitro Experiments

To create constructs for overexpression of IGF-1 in vitro, SP1-containing IGF-1 cDNAs were cloned into the Sal1/BamH1 backbone of pIRES2-EGFP. The cDNA encoding mature IGF-1 lacking E-peptides was denoted IGF-1-stop. To prevent cleavage of the E peptide moiety, the cleavage site in IGF-1Ea and IGF-1Eb cDNAs was mutated (RS71/72 deletion) using site directed mutagenesis to generate cleavage-defective isoforms - IGF-1EaCD and IGF-1EbCD respectively. All cDNAs included the SP1 signal peptide.

To generate the relaxin expression constructs, RLN1-V5/His,RLN1-Ea-V5/His, RLN1-Eb-V5/His, cDNA of mouse relaxin was cloned into pcDNA3.1-V5/His vector (Invitrogen), E-peptides were added using the QuikChange method (Stratagene).

### Cell Cultures and Transfection

HEK 293 cells were cultured in growth medium (DMEM supplemented with 10% fetal bovine serum (FBS), 2 mM L-glutamine, 1 mM Na-pyrovate, 10 mM HEPES and 1× NEAA (all from Gibco/Invitrogen). Transient transfections were performed with Lipofectamine™ 2000 (Invitrogen) according to the manufacturer’s instructions. Medium was harvested 24–30 hours after transfection.

### Immunoenzymometric Assay (IEMA)

To determine circulating IGF-1 levels and IGF-1 levels in the conditioned growth media, OCTEIA Rat/Mouse IGF-1 IEMA (iDS) was used according to the manufacturer’s instructions.

### Binding to Tissue Culture Surfaces and Heparin Agarose

BD PureCoat plates (Carboxyl – negatively charged and Amine – positively charge), and immobilized heparine (Thermo Scientific, 20207) and control agarose beads (Thermo Scientific, 26150) were used for in vitro binding experiments. 500 uL of conditioned growth medium (with IGF-1 levels normalized to 200 ng/mL) was incubated in the wells of the tissue culture plates or with agarose beads for 1 hour at 37C. The plates or agarose beads were then washed 3 times with PBS and the bound proteins extracted with 50 ml 1× SDS loading buffer.

### Immunoblotting

Protein extracts from mouse tissues were prepared in RIPA lysis buffer (20 mM Tris pH 8.0, 5 mM MgCl_2_, 150 mM NaCl, 1% NP40, 0.1% Triton X, 1 mM NaVO_4_, 1 mM NaF, 1 mM PMSF, 1 ug/ml of Aprotinin and Leopeptin). 30–50 ug of protein lysates were used for each sample, separated by SDS-page, and immunoblotted. Filters were blocked in 5% milk (Roth, T145.1) in TBST (20 mM Tris pH 7.5, 140 mM NaCl, 0.1% Tween20). Primary and secondary antibodies were diluted in blocking solution according to the manufacturer’s suggestion. Primary antibodies used: anti mouse-IGF-1 (Sigma, I-8773), anti V5 SV5-Pk1 (abcam, ab27671); secondary antibodies used: donkey anti-goat IgG-HRP (Santa Cruz, sc-2020), sheep anti-mouse IgG-HRP (GE Healthcare, NA931).

### Decellularization

Lung and skeletal muscle tissue (quadriceps) was removed from two month old male C57BL/6 mice sacrificed by cervical dislocation. The tissue was rinsed in PBS and decellularized as described by [23]. Briefly, the tissues were treated with latrunculin B and subsequently incubated in high salt solutions to disrupt the cells by osmotic shock. After DNase treatment, the decellularized tissues were washed in water for 2 days.

### Histology

For preparation of paraffin sections, tissues were fixed in Modified Davidson’s Fixative (Polysciences, 24355), dehydrated in series of ethanol dilutions, passed though xylene, xylene/paraffin, and embedded in paraffin. Sections were cut at 10 um and stained with Haematoxylin/Eosin or DAPI.

### Binding to ECM

500 uL of media from transfected HEK293 cells was supplemented with proteinase inhibitors (Roche, 04693132001) and incubated with 75 mg (wet weight) of decellularized tissue for 1 hour at RT. After incubation samples of the media were taken to test for protein degradation during incubation. The tissue was then washed three times 30 minutes with PBS to remove unbound peptides. Bound peptides were then eluted from the decellularized tissue using 50 uL of standard Laemmli SDS-loading buffer. 20 uL of media that had not been incubated with decellularized tissue, 20 µL of the media sample taken after incubation, and 40 µL of the eluate were loaded on an 18% polyacrylamide gel and IGF-1 (Figure 6) or relaxin (Figure 8) was detected by Western blot. In the case of IGF-1, the primary antibody was a goat anti-IGF-1 antibody (Sigma, I-8773) was used at a 1∶1000 dilution and the secondary antibody, donkey anti-goat IgG-HRP (Santa Cruz, sc-2020) was used at 1∶10.000. In the case of relaxin, the peptide was detected using a primary antibody against the V5 tag (abcam, ab27671) and a mouse secondary antibody, sheep anti-mouse IgG-HRP (GE Healthcare, NXA931) at a dilution of 1∶10.000.

### Immunohistochemistry

IGF-1 concentrations in media from HEK 293 cells transfected with IGF-1 encoding constructs were determined and the amount of IGF-1 was adjusted (using media from untransfected cells) to 200 ng/mL. Sections of decellularized tissue were deparaffinised using xylene and rehydrated by passaging through solutions of decreasing ethanol concentrations, then incubated in the normalized IGF-1 conditioned media. After removal of the media, the sections were washed and fixed in 4% formaldehyde, blocked in 5% normal donkey serum, and incubated with the primary antibody (goat anti-mouse-IGF-1 (Sigma, I-8773) at 1∶6 dilution in blocking buffer at 4C overnight). Subsequently, the sections were incubated with biotinylated horse anti-goat IgG antibody (Vector Labs, BA-9500) at 1∶200 dilution at 4C for 48–72 hrs followed by incubation with streptavidin-Alexa 594 (Invitrogen, S-11227) for 1hr at RT. Images was acquired with Leica TCS SP5 confocal microscope, and the number of foci was counted using ImageJ software.

### Deglycosylation of IGF-1 Propeptides

Conditioned growth medium (with IGF-1 levels normalized to 200 ng/mL) was deglycosylated in non-denaturing reaction conditions for 4 hours at 37C using Protein Deglycosylation Mix (NEB, P6039) (1 ul of the mix per 10 ul of conditioned growth medium).

### Statistical Analysis

Data is presented in mean±S.D. The Student’s t test (tail 2, type 2) was used for statistical analysis and differences were considered significant when p-value was <0.01.

## Supporting Information

Figure S1 **Characterization of skeletal muscle specific IGF-1 transgenic mouse lines.** (A) Transgene expression in founder MLC/IGF-1 lines assessed by Northern blot analysis on 10 ug of total RNA of each tissue with a probe specific for the SV40 polyadenylation sequence. Transgenic samples of every line were compared to WT littermates. (B) Comparison of transgene expression levels between the four MLC/IGF-1 lines by Northern blot analysis on 10 ug of total RNA from quadriceps muscle using a SV40-specific probe.
(TIF)

Figure S2 **Deglycosylation of IGF-1 propeptides increase their affinity to negatively charged surfaces.** A) Growth medium from transiently transfected HEK 293 cells incubated with (lanes 2 and 4) and without (lanes 1 and 3) deglycosylation enzyme mix (IGF-1 levels normalised to 200 ng/mL; 20µl load). B) Binding of deglycosylated (lanes 6 and 8) and non-deglycosylated (lanes 5 and 7) IGF-1 propeptides to negatively charged tissue culture plates.
(TIF)
