# Supplementary figures and images for: E-Peptides Control Bioavailability of IGF-1
Source: PLoS One. 2012 Dec 10;7(12):e51152. doi: 10.1371/journal.pone.0051152 (PMC3519493; doi:10.1371/journal.pone.0051152)

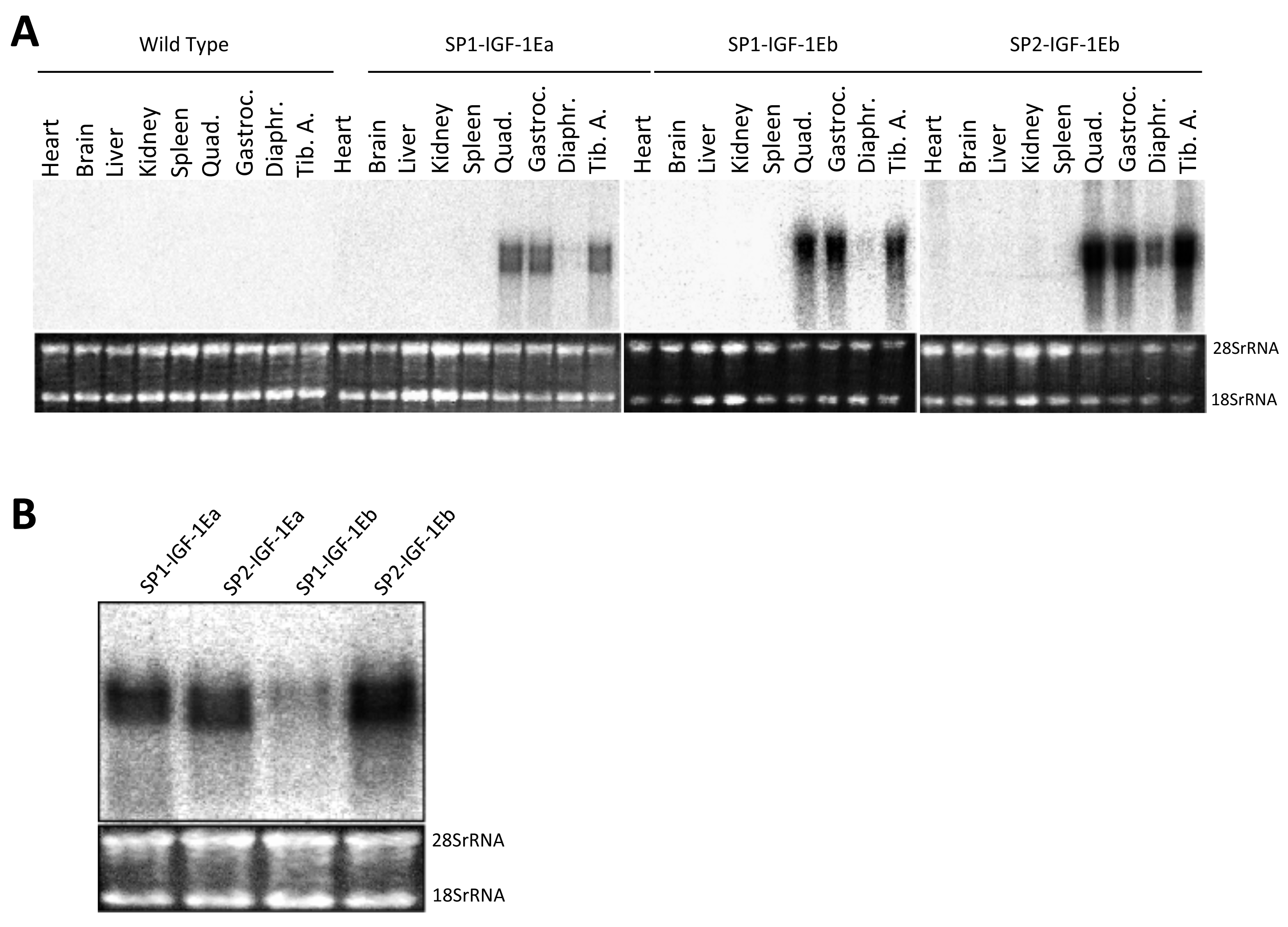

Supplement: Figure S1 — Characterization of skeletal muscle specific IGF-1 transgenic mouse lines. (A) Transgene expression in founder MLC/IGF-1 lines assessed by Northern blot analysis on 10 ug of total RNA of each tissue with a probe specific for the SV40 polyadenylation sequence. Transgenic samples of every line were compared to WT littermates. (B) Comparison of transgene expression levels between the four MLC/IGF-1 lines by Northern blot analysis on 10 ug of total RNA from quadriceps muscle using a SV40-specific probe. (TIF) [file pone.0051152.s001.tif]

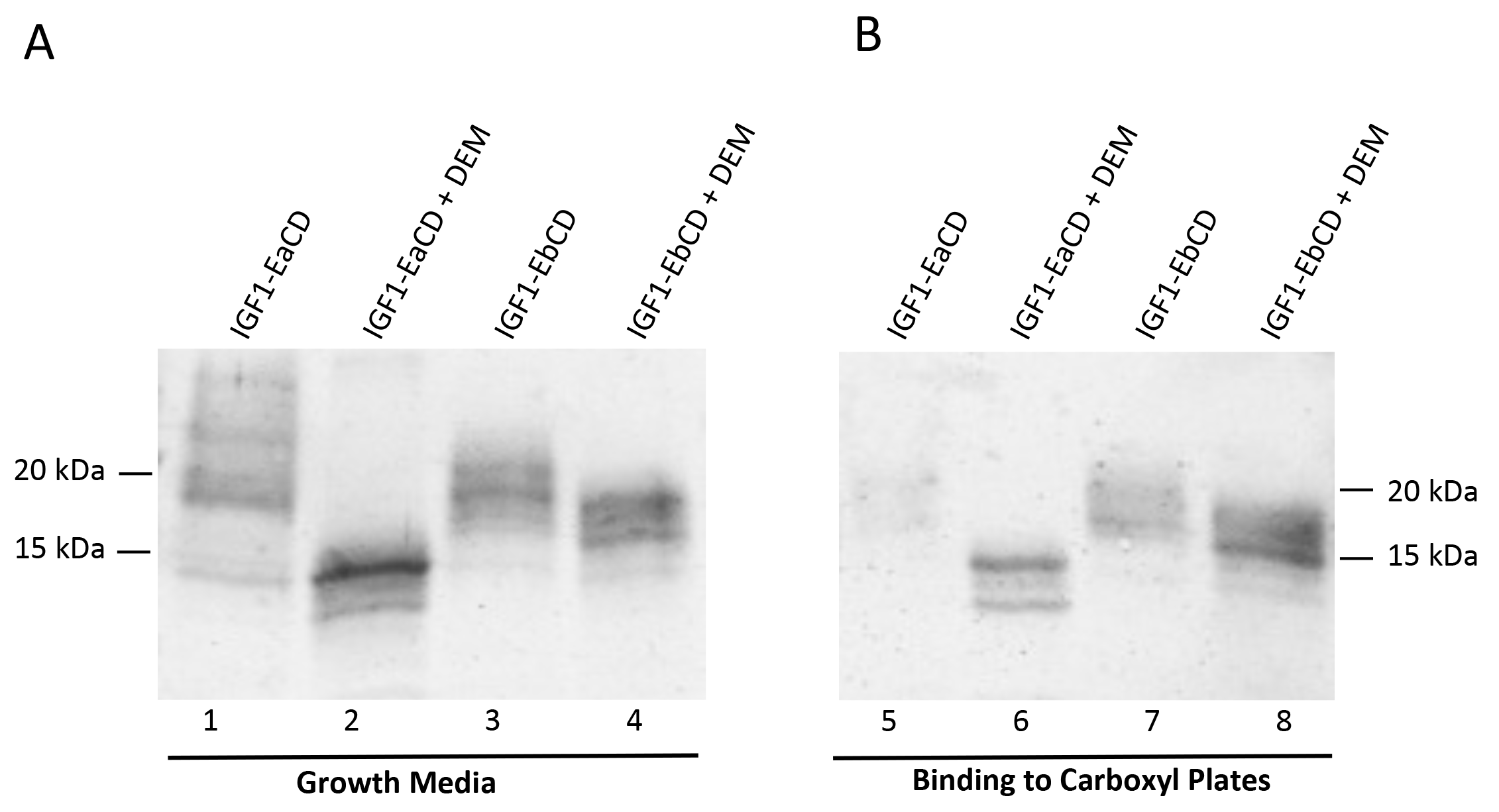

Supplement: Figure S2 — Deglycosylation of IGF-1 propeptides increase their affinity to negatively charged surfaces. A) Growth medium from transiently transfected HEK 293 cells incubated with (lanes 2 and 4) and without (lanes 1 and 3) deglycosylation enzyme mix (IGF-1 levels normalised to 200 ng/mL; 20µl load). B) Binding of deglycosylated (lanes 6 and 8) and non-deglycosylated (lanes 5 and 7) IGF-1 propeptides to negatively charged tissue culture plates. (TIF) [file pone.0051152.s002.tif]
